# Do the Types of Dietary Carbohydrate and Protein Affect Postprandial Glycemia in Type 1 Diabetes?

**DOI:** 10.3390/nu17111868

**Published:** 2025-05-29

**Authors:** Xinyi Li, Alice Wainwright, Chantelle Z. Fio, Shannon Brodie, Kylie Alexander, Margaret McGill, Sally-Anne Duke, Gregory Fulcher, Stephen Twigg, Jencia Wong, Jennie Brand-Miller, Garry M. Steil, Kirstine J. Bell

**Affiliations:** 1 Charles Perkins Centre, The University of Sydney, Sydney 2006, Australia; 2 The Children’s Hospital at Westmead Clinical School, Faculty of Medicine and Health, The University of Sydney, Sydney 2006, Australia; 3 Royal North Shore Hospital Diabetes Centre, Sydney 2065, Australia; 4 Royal Prince Alfred Hospital Diabetes Centre, Sydney 2050, Australia; 5 Harvard Medical School, Boston, MA 02115, USA; 6 Boston Children’s Hospital, Boston, MA 02115, USA

**Keywords:** type 1 diabetes, insulin dosing, postprandial glycemia, protein, carbohydrate

## Abstract

**Background/Objectives**: Dietary protein and carbohydrate affect postprandial glycemia in individuals with type 1 diabetes (T1D). This paper aimed to determine the relationship between the types of dietary protein (Study 1) and carbohydrate (glycemic index; GI, Study 2) and postprandial glycemia. **Methods**: Two acute randomized crossover trials were conducted in adults with T1D comparing postprandial glycemia for test meals varying by protein type (*n* = 16 adults; 5 meals: egg, beef, chicken, salmon or whey (all 30 g protein), each served with fried rice (45 g carbohydrate) or GI (*n* = 8 adults, high or low GI bread, GI 52% vs. 76%) with peanut butter (19 g protein, 30 g fat). Insulin was dosed based on usual individualized insulin: carbohydrate ratio and capillary blood glucose levels (BGL) measured from 30 min pre- to 5 h postprandially in 15–30 min intervals. **Results**: Study 1: Postprandial glycemia varied over an almost 2-fold range, however responses were highly variable and there were no significant differences between sources (iAUCglucose Chicken: 203 ± 66 mmol·min/L, Egg: 263 ± 100 mmol·min/L, Beef: 309 ± 89 mmol·min/L, Salmon: 338 ± 83 mmol·min/L and Whey: 397 ± 115 mmol·min/L respectively, *p* > 0.05). Hypoglycemia (≤3.5 mmol/L) occurred at least once per protein type (chicken: 6/16 participants, egg 2/16, beef 3/16, salmon 1/16, whey 2/16). However, there were no statistically significant differences in the risk of hypoglycemia between protein sources (*p* > 0.05). Study 2: Postprandial glucose response curves were virtually identical for high GI and low GI, and the incremental area under the curve (iAUC) for glucose was not statistically significant after 1 h (*p* = 0.185), 3 h (*p* = 0.538) or 5 h (*p* = 0.694) following the meal. **Conclusions**: Clinical practice guidelines and insulin dosing algorithms likely do not need to consider differences in protein sources or in GI in the context of a high fat, high protein meals, for individuals with T1D.

## 1. Introduction

Type 1 diabetes (T1D) is an incurable autoimmune condition. Management is complex, burdensome and costly, revolving around intensive glucose monitoring and multiple daily injections or continuous insulin infusions to maintain blood glucose levels within the narrow healthy range [1]. Maintaining glycemic control is essential to prevent acute and long-term complications of diabetes, including hypoglycemia, retinopathy, neuropathy and cardiovascular events [1]. Mealtime insulin dosing based on the amount of carbohydrate consumed is the gold standard approach, however following a convincing body of evidence, current clinical guidelines now also advocate for protein and fat to be considered in the mealtime dosing algorithm, as well as nutrition education focused on dietary quality [1,2].

Fat and protein have been shown to have notable effects on postprandial glucose levels and insulin requirements in several studies [3,4,5,6,7,8,9]. We have previously shown that the glucose area under the curve (iAUC) more than doubled with the addition of 40 g of fat and 27 g of protein, despite the same carbohydrate content and insulin dose [4]. Studies have also demonstrated dietary fat significantly reduces the early glucose response from 0–3 h and delays the glucose peak [3,10]. In contrast, high fat, high protein meals increase the postprandial glycemic response significantly in the late postprandial period, from 3 h onwards after meal [4,11]. The effect of consuming fat and protein together on glycemia has been shown to be additive [7]. The different insulinotropic effects between protein types seen in other populations provides reason to believe that the type of protein may also influence glycemia and insulin requirements in T1D and therefore warrants investigation. For example, von Post-Skagegård et al. [12], found significant differences in both glucose and insulin responses to 3 different proteins (cottage cheese, soy protein isolate and lean cod fish) in 17 healthy women, despite matched energy and macronutrient contents [12]. The Food Insulin Index (FII), which summarizes the relative postprandial insulin secretion to single foods in healthy adults, also suggests the type of protein may impact glycemia [13,14]. FII testing has shown white fish (FII: 46%) stimulates almost twice as much an insulin secretion as an isoenergetic portion of poached eggs (FII: 26%) and more than five times as much insulin as an isoenergetic portion of bacon (FII: 9%) [13].

Whilst the amount of carbohydrate consumed is the predominant driver of postprandial glycemia, the carbohydrate quality, or glycemic index (GI) is also known to impact glucose control. The GI is a relative, quantitative measure ranking carbohydrate-rich foods based on their impact on postprandial glycemia [15]. Low GI carbohydrate foods are metabolized more slowly and produce a lower peak glucose compared with the characteristic rapid glucose spike seen with high GI carbohydrates [15]. Meta-analyses have shown that a low GI diet may help to reduce HbA1c and fasting glucose for those living with diabetes [16,17,18] and healthy, low GI carbohydrate foods are recommended for T1D in international clinical practice guidelines [2,19]. A 2023 review has also highlighted the improvement in glycemic control with low-GI diets in children with T1D. However, it was suggested limiting, rather than excluding, high and moderate-GI foods may improve sustainability of the diet [20]. The acute glycemic impact of GI has also been demonstrated in T1D, with a high GI meal resulting in a significantly higher postprandial glycemic response between 30 and 180 min compared to low GI meal in 20 children and adolescents [21]. However questions have arisen regarding the impact of varying the GI, especially in the context of high fat and protein meals where carbohydrate amount and type are usually held constant, and the subsequent implications for insulin dosing [3,4,10,19].

Despite GI, fat and protein all being known to impact the early postprandial glycemic response individually, the glycemic impact of varying the type, their combined impact and the subsequent implications for insulin dosing in T1D remain unclear. Therefore, in order to provide evidence-based guidance on insulin dosing for meals, it is essential to understand whether the type of protein and/or carbohydrate, in the context of a mixed meal, will influence blood glucose levels differently and thus impact insulin dosing algorithms. Specifically, Study 1 aimed to compare the relationship between postprandial glycemia and five commonly-consumed protein foods (beef, chicken, salmon, eggs and whey protein) in adults with T1D. Study 2 aimed to compare the impact a high GI carbohydrate vs. a low GI carbohydrate on the postprandial glycemic response in the context of a high fat, high protein, low-moderate carbohydrate mixed meal in adults with T1D.

## 2. Materials and Methods

Two independent, acute, randomized within-subject trials were conducted in adults with T1D, comparing the impacts of five protein types (Study 1; 2018–2019 and 2019–2020: recruitment paused due to COVID-19 restrictions) and two carbohydrate types (i.e., differing in GI; Study 2; 2020–2021) on postprandial glycemia. The studies were conducted at the Charles Perkins Centre Research Facility at the University of Sydney. Ethics approval was obtained from the Sydney Local Health District Human Research Ethics Committee and the studies were registered (Study 1: ACTRN12618001344280 Study 2: ACTRN12621000081819), Written informed consent was obtained for all participants.

Participants were recruited through advertising via posters around the University of Sydney, an existing clinical research database, T1D trial registers and flyers on social media. The eligibility criteria was the same for both studies: aged between 18–70 years inclusive, T1D diagnosis for greater than or equal to 1 year, HbA1c less than or equal to 8.5%, reliably self-monitoring blood glucose at least 4 times per day/or using continuous glucose monitoring and fluency in English. Exclusion criteria included concurrent medical issues including coeliac disease, gastroparesis, food allergies, intolerances or eating disorders or use of other medication that may influence blood glucose levels, pregnancy or lactation. All participants underwent a venous blood test to assess their HbA1c, lipids, C-peptide and C-reactive protein to confirm eligibility and assess baseline demographics.

Study sessions were conducted on separate mornings, with sessions permitted on consecutive days. On the day prior to each study session, participants were instructed to avoid alcohol, exercise, legumes and high fat foods and to fast from midnight (water accepted). Participants we also asked to minimize insulin adjustments overnight and avoid all manual insulin adjustments in the 3-h prior to the session. In the case of overnight or morning hypoglycemia, participants were instructed to treat according to their usual care and the session was rescheduled.

On the morning of each test session, fasting blood glucose levels were required to be between 4–11 mmol/L (Study 1) or 4–10 mmol/L (Study 2) to commence the study session. Sessions for Study 1 were conducted on-site at the Charles Perkins Centre OR supervised sessions in participants’ home (modification due to COVID-19 pandemic), whilst Study 2 sessions were all conducted from participants’ home with remote monitoring. During each study session, glucose monitoring began 30 min prior to the meal (t = −30 min). After 15 min (t = −15 min), each individual self-administered their rapid-acting insulin dose based on their prescribed individualized insulin: carbohydrate ratio (identical dose for all sessions, with no correction for fasting glucose). A further 15 min later (t = 0 min), they consumed their allocated meal. Participants using ultra-fast acting insulin self-administered their insulin dose immediately prior to their allocated meal (t = 0 min). The order of test meals was randomized using a computer-generated sequence, participants were randomized sequentially in the order they provided informed consent and the allocation order was not concealed from the investigators, to allow meal preparation. In Study 1, blinding of participants was not possible due to the visible differences in the test meals, whereas in Study 2, participants were blinded to their allocated test meal. All test meals were served with 250 mL of plain water and participants were given 12 min to consume both the food and water and then no other food or drink allowed for the remainder of the test session (except water from 60 min after meals). Glucose levels were monitoring for 5 h postprandially and participants were instructed to remain sedentary for the full 5.5 h study period. If hypoglycemia occurred (<3.5 mmol/L), test sessions were terminated, participants treated their glucose level according to their usual procedure and the event was recorded.

### 2.1. Test Meals

Test meals were prepared by nutrition research team and ingredients were weighed to the nearest 0.1 g using Electus kitchen scale (Model QM-7264; Electus, Rydalmere, Australia) in the onsite metabolic kitchen. 

Study 1 (Protein): The five protein test meals consisted of one of five sources of protein; egg, chicken, beef, salmon or whey isolate, served with fried rice (white rice, peas, corn, carrot and soy sauce; Supplementary Material Table S1). All five test meals were matched for carbohydrate (45 g) and protein (30 g). The meals were all pre-prepared and frozen for standardization. On the day of testing, meals were defrosted and reheated in a frypan. Salmon was cooked from raw (frozen) on the day of testing.

Study 2 (GI): The two GI test meals consisted of toasted white bread, varying in GI, served with peanut butter. Both meals provided 37 g of carbohydrate, 30 g of fat and 19 g of protein (Supplementary Material Table S2) and varied only in the GI of the bread (high GI (76%) vs. low GI (52%). Test breads were provided frozen for freshness and standardization and were individually wrapped and labelled as ‘Meal A’ and ‘Meal B’ and the order they were to be consumed. On the day of each session, participants were instructed defrost and lightly toast the allocated bread before adding the full serve of peanut butter.

### 2.2. Glucose Monitoring

Study 1 (Protein): Capillary blood glucose levels were analyzed using a Hemo-Cue Glucose 201+ analyzer. Samples were collected at t= −30 min, −15 min and 0 min, as above, and then subsequently monitored at t = 15 min, 30 min, 45 min, 1 h, 1.5 h, 2 h, 2.5 h, 3 h, 3.5 h, 4 h, 4.5 h and 5 h.

Study 2 (GI): Interstitial glucose levels were monitored using flash glucose monitoring (FGM, Abbott Freestyle Libre, Victoria, Australia), taking advantage of the flexibility and increasing affordability of this newer technology and allowing the study to be conducted remotely. Both test sessions were completed within the one 14 day period (maximum FGM duration), excluding the first 24 h of starting FGM to reduce transient inflammation and improve FGM accuracy. The FGM system recorded the interstitial blood glucose level every 15 min, and data was extracted for 30 min prior to the meals and for 5 h postprandially.

### 2.3. Statistical Analysis

The primary outcome for both studies was the difference in the glucose incremental area under the curve (iAUC) between test meals over 5 h (Study 1) and 3 h (Study 2), reflecting the timing of impact of the nutrients on blood glucose levels in the literature [10]. Secondary outcomes were 0–2 h iAUCglucose (early postprandial period), 2–5 h iAUCglucose (late postprandial period), mean glucose level, standard deviation (SD) around mean glucose level, coefficient of variation (CV), J-Index (J = 0.324 * (mean glucose level + SD) 2), peak glucose level, time to peak glucose level, nadir glucose level, time to nadir glucose level, and amplitude (equal to peak minus nadir glucose level). Differences in all metrics were assessed using; Study 1: one-way repeated measures ANOVA and Study 2: paired *t*-test (GraphPad Prism, version 9.0.1). In addition to the planned comparisons in Study 1, a post-hoc exploratory mixed modeling analysis was performed on the primary outcome using both fasting glucose and protein type as variant (STATA (2021, Version 17). Post-hoc power calculations were performed for Study 1 using MLAB (Civilized Software) to simulate data sets (*n* = 100). In the case of early termination/hypoglycemia (<3.5 mmol/L), the last recorded value was carried forward and included in the analysis. The proportion of hypoglycemia episodes was analyzed using a Kaplan-Meyer survival plot (Supplementary Material Figure S1). Data are expressed as mean ± SEM unless otherwise stated. Differences were considered statistically significant if the *p*-value was <0.05 and highly significant if the *p*-value was <0.01 (two tailed).

Sample Size: Study 1: Preliminary power calculations, in which the total effect size was equally spread across the 5 test meals, and the variability of the effect (standard SD) was assumed to be 1.25 times the effect size, indicated 16 subjects would provide 80% power to detect differences in 5 h iAUCglucose (duration of effect of protein). Study 2: The study was powered to detect a difference in 3 h iAUCglucose of 100 mmol·min/L assuming a SD of 75 mmol·min/L (duration of effect of carbohydrate). Seven participants provided 80% power, with the final sample size increased to 10 participants to allow for dropout.

## 3. Results

### 3.1. Study 1: Protein Type

Sixteen adults met the inclusion criteria and were enrolled in the study. The mean age of participants was 35.5 ± 18.2 (range 19–64) years and 75% were female (Table 1). Fourteen participants (88%) used insulin pump therapy and 2 participants (12%) used MDI. The mean duration of diabetes was 16.8 ± 9.6 years and mean HbA1c was 7.4 ± 1.2% (57 ± 11 mmol/mol). All participants used a rapid acting insulin analog (Insulin Aspart or Insulin Lispro). Mean meal insulin dose was 6.0 ± 2.2 units.

**Figure 1:**
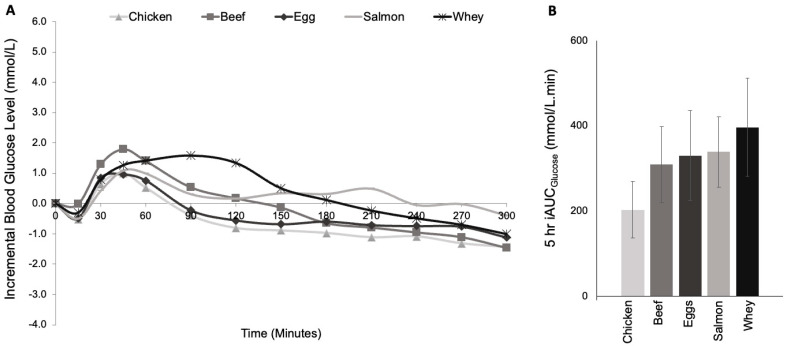
Mean blood glucose levels (**A**) and Incremental area under the curve (iAUC) for blood glucose over 5 h (**B**) following 5 protein types (egg, beef, chicken, salmon and whey) with identical carbohydrate content in adults with type 1 diabetes (*n* = 16).

No significant differences in 5 h iAUCglucose were observed among the meals (203 ± 66 mmol·min/L, 263 ± 100 mmol·min/L, 309 ± 89 mmol·min/L, 338 ± 83 mmol·min/L and 397 ± 115 mmol·min/L for chicken, egg, beef, salmon and whey respectively, *p* = 0.347, Table 2, Figure 1), and, despite the almost 2-fold higher iAUCglucose (96%) for chicken vs. whey, the difference was not significant by post-hoc paired *t*-test, *p* = 0.096). There were no differences in early (0–2 h) or late (2–5 h) iAUCglucose (Table 2, Figure 1). There was considerable intra-individual variability in glycemic responses, with the within-individual standard deviation in 5 h iAUCglucose varying over a 20-fold range (range: 24–496 mmol·min/L).

Glycemic variability assessed as SD, CV, or amplitude was also not different (Table 2; *p* > 0.05 all). In contrast, there was a significant difference in J-index, a measure of glycemic variability incorporating both the glucose mean and SD (*p* = 0.028). The incremental peak glucose level ranged from 2.2 ± 0.4 mmol/L (chicken) to 3.0 ± 0.5 mmol/L (whey, *p* > 0.05) and the time to peak ranged from 74 ± 19 min [22] to 133 ± 25 min [23]. The glucose level ranged from −2.6 ± 0.6 mmol/L [22] to −2.1 ± 0.4 mmol/L (salmon, *p* > 0.05) and the time to nadir ranged from 118 ± 24 min (chicken) to 142 min (whey, *p* > 0.05). Hypoglycemia (<3.5 mmol/L) occurred in 1 or more participants for each protein source during the 5 h test (salmon: 1/16 participants, whey: 2/16, egg: 2/16, beef: 3/16, chicken: 6/16; not different, *p* > 0.05).

Fasting glucose was similar in all groups (*p* = 0.198). However, the differences that were observed (range 7.4 to 8.6 mmol/L, 16% difference) had a significant effect on 5 h iAUCglucose when assessed using a post-hoc mixed modelling analysis (effect of fasting glucose on iAUCglucose significant, *p* = 0.005, with no effect of protein type on iAUCglucose; *p* = 0.091; Table 2). The effect of protein type was not significant by either repeated measures ANOVA analysis (*p* = 0.347) nor by mixed model analysis with fasting glucose as a variable. Retrospective power analysis using the measured effect sizes and variability indicated the predefined repeated measures analysis did not have sufficient power to detect differences and that 51 participants would be needed to obtain 80% power.

### 3.2. Study 2: GI Study

Ten adults with T1D (3 men, 7 women) were recruited, however two participants were excluded due to protocol violations; one did not meet the inclusion criteria after commencing test sessions (HbA1c above inclusion criteria) and the other participant had given a substantial insulin correction dose 3 h prior to the session, which had lowered glycemia by ~10 mmol/L leading up to the start of the session. There was no postprandial glucose rise during the test session (t = −30 min highest glucose value), indicating they were substantially overinsulinized during the test period. Mean results of the remaining 8 participants are presented. The mean age was 29.0 ± 6.0 years, BMI 26.2 ± 7.2 kg/m^2^, time since diagnosis 18.0 ± 6.8 years, HbA1C was 7.5 ± 0.7% (59 ± 8 mmol/mol), and mean mealtime insulin dose was 4.7 ± 0.8 units (identical for both meals; Table 1). Two participants used ultra fast-acting insulin (Fiasp) and the remaining participants (n= 6) used a rapid-acting insulin (Novorapid).

**Figure 2:**
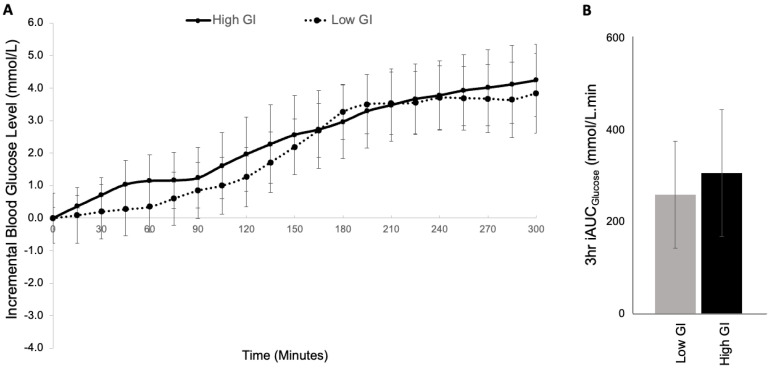
Mean blood glucose levels (**A**) and Incremental area under the curve (iAUC) for blood glucose over 3 h (**B**) following a high GI vs. Low GI carbohydrate source within an identical high fat, high protein mixed meal in adults with type 1 diabetes (*n* = 8).

As intended, mean fasting glucose was similar at the start of both meals (high GI: 7.6 ± 0.4 mmol/L vs. low GI: 7.9 ± 0.9 mmol/L; *p* = 0.766; Figure 2, Table 3). The postprandial glucose response curves were virtually identical and the incremental area under the curve (iAUC) for glucose was not statistically significant after 1 h (*p*= 0.185), 3 h (*p* = 0.538) or 5 h (*p* = 0.694) following the meal. There were no statistical differences in the incremental mean glucose level (high GI: 2.1 ± 0.6 mmol/L vs. low GI: 1.9 ± 0.5 mmol/L; *p* = 0.646). Similarly, there were no significant differences in the postprandial glucose variability between high and low GI test meals, including in the incremental peak BGL (*p* = 0.777), time to peak (*p* = 0.231) and incremental amplitude (*p* = 0.580). There were no hypoglycemic events recorded following either of the test meals in any participants.

## 4. Discussion

To our knowledge, these two studies are the first to examine whether the types of protein and/or carbohydrate in the context of a mixed meal influence the postprandial glycemic response in individuals with T1D. The results of both studies show that in adults with T1D, consuming different protein or carbohydrate types in mixed meals have a small, highly variable and clinically and statistically insignificant impact on postprandial glycemia. However, these negative findings still address important clinical questions and address gaps in the literature on the impact of macronutrient types, as identified in an influential paper on the topic [10]. Specifically, these studies illustrate that individuals with T1D likely do not need routine education to account for different protein sources or the GI of the carbohydrate in the context of high fat, high protein mixed meals in their mealtime insulin doses. Furthermore, researchers and developers working on new bolus estimators likely do not need to add complicated corrections for GI or protein source into their algorithms.

The foundational premise of the GI is that it influences the postprandial glycemic response. Considering the result of Study 2, we are not suggesting that GI has no clinical relevance in T1D, but rather that high proportions of fat and protein, relative to carbohydrate, mask the impact of the GI on postprandial glycemia in adults with T1D. When test meals are relatively low in fat and protein, the GI has been shown to significantly impact glycemia in T1D. For example, Ryan et al. [21], demonstrated a significant higher postprandial glycemic response to a high vs. low GI meal in children with T1D. However, these test meals were 2–3 times lower in fat and protein (~10 g each) compared with Study 2 (~30 g and ~20 g respectively). Similarly, even when meals were higher in fat and protein (27–44 g and 14–38 g respectively), but carbohydrate was also very high (~50–130 g carbohydrate/meal, ~150–350% higher than Study 1), the GI has been shown to alter the glycemic response in T1D [24,25,26,27,28,29]. However, many of these studies did not control for the macronutrient contents, ingredients and/or insulin dosing, thereby confounding direct comparison.

The lack of power to detect differences in meal responses, particularly in Study 1 where there was a 2-fold difference between protein types, could be attributed to the high variability in meal responses between participants. Indeed, our results show that the SD in the protein meal responses were roughly 4 times greater than the observed glycemic differences, despite strict diet and exercise instructions on the day prior to the meals and 30 min glycemic monitoring prior to each test meal. However, with the realistic meals used in these studies, the meal effect sizes were relatively small. The protein content could be increased in a future trial to increase the effect size and thus the study power, however this may not be practical (i.e., 30 g protein from eggs equated to almost 5 large eggs, so scope for increasing protein content is very limited for some foods). Furthermore, post hoc power analysis of the current trial indicates 51 participants would need to be conducted to ascertain whether there is a significant effect on glycemia by animal protein type. Thus, while nutrient effects could potentially be shown to be statistically significant in a substantially larger trial, it is not clear that such a trial would be clinically valuable (i.e., unlikely to result in routine advice for insulin adjustment for usual meals in clinical practice) and is thus unlikely to be warranted. Insulin dose adjustments may still be appropriate, however, in a subset of individuals with high variability between meals if their results can be shown to be consistently reproducible.

Our findings also suggest that other factors affecting the meal response, such as the effect of fasting glucose, should be looked at more closely. Mixed model analysis, including both animal protein type and fasting glucose as predictors, showed that relatively small changes in fasting glucose (<16% on average; NS) can yield substantial, statistically significant changes in iAUC. This is an important finding and raises an interesting research and clinical question. The result can potentially be explained by acute changes in insulin sensitivity. The repeated measures analyses conducted here used incremental response data, which should, in theory, correct for any differences in fasting glucose. However, under fasting conditions, a significant portion of total glucose uptake is thought to be insulin independent, suggesting that a relatively small increase in fasting glucose could be indicative of a much larger decrease in insulin sensitivity [30]. Indeed, using the data from Bergman et al., reducing insulin sensitivity by 50% only equates to a 20% increase in fasting glucose. However, a 50% decrease in insulin sensitivity would, in theory, require a 100% increase in the mealtime insulin bolus [30]. This is similar to the finding in our present study, where a 16% difference in fasting glucose was a significant predictor of the 96% increase in glucose response (Table 2). If so, current approaches for adjusting meal insulin coverage when the preprandial glucose level is above target may need to be revaluated.

The findings are logical and consistent with the physiological responses to nutrients. Both fat and protein are known to delay gastric emptying [11], which alters the appearance of glucose in the bloodstream. Any difference in glucose appearance due to the GI of the carbohydrate would likely be overwhelmed by the presence of fat and protein. This effect is sometimes seen in clinical recommendations, whereby fat and protein is used to try to reduce the postprandial glycemic spike associated with high GI meals [2]. It was interesting to see in Study 2 that there was neither a pronounced glycemic spike with the high GI carbohydrate meal nor an increased risk of early hypoglycemia with the low GI carbohydrate meal. Our findings are also consistent with our previous study showing postprandial glycemia and insulin requirements differed by dietary fat amount but not type [3].

Our studies have several strengths. The 30 min ‘run-in’ time confirmed glycemic stability prior to the meal and glycemia was monitored for 5 h postprandially to assess the delayed impact of protein and/or fat. Both studies included both pump therapy and MDI participants (though the vast majority used pump therapy) and thus results are applicable to both patient groups in clinical practice. In addition, the test meals reflect realistic meals, using commonly consumed animal protein sources for Study 1, and common breakfast foods (toast and peanut butter) for Study 2. Test meals for Study 1 contained 30 g protein, more than twice the amount of protein shown to induce a statistically significant impact on glycemia when consumed with carbohydrate [31] and fat and protein have been shown to have an additive impact when consumed together [7]. The study design also intentionally included the identical protein source (whey protein isolate) used in many of the leading T1D protein studies [31,32], and thereby validates that the results seen in those studies can likely be extrapolated to other animal protein types. The test meals were highly controlled, with the ingredients, energy, macronutrient and fiber contents of the test meals matched, wherever possible, to facilitate accurate comparison. For Study 2, the only difference was in the GI of the carbohydrate (white bread) and there was a greater than 20 percentage point difference between the two carbohydrate sources (52% vs. 76%).

There are also limitations to both studies. The number of participants was small, but this is not unusual for these types of highly controlled, acute meal studies. As we included both pump and MDI in the inclusion criteria, insulin was given as a normal bolus rather than a dual-wave insulin pattern, which has previously been demonstrated to be better suited to high fat and/or protein meals [19]. However, both insulin dose and delivery pattern were kept consistent between meals and participants to facilitate direct comparison. We did not include a run-in period to optimize insulin therapy prior to study sessions of Study 2, however we set an HbA1c upper threshold as part of the inclusion criteria, used individualized insulin: carbohydrate ratios, matched both basal and bolus insulin therapy across both sessions and had a strict protocol for 24 h prior to each session to reduce interindividual variability. In terms of test meals, in Study 1, meals were matched for protein and carbohydrate contents but could not be matched for energy and fat contents because these vary intrinsically with protein source. Dietary fat is known to also impact postprandial glycemia [10,33], however this is unlikely to account for the 2-fold difference in iAUC observed, given the protein types inducing both the highest and lowest glucose responses contain just 0–2 g of dietary fat (whey and chicken). Also, Study 1 only investigated animal protein sources and it is possible that plant-based protein sources may impact glycemic responses differently. Thirdly, the study cannot rule out the possibility that specific individuals may have consistently higher or lower glycemic responses to different animal protein sources. As highlighted earlier, clinically, it may be reasonable to adjust insulin doses for protein source for individuals with a clinically relevant and consistently reproducible effect by protein type. Our findings question the need for a larger trial, as high variability relative to the effect size limits both the feasibility and clinical relevance. Future studies using closed-loop insulin systems, or hyperinsulinemic euglycemic clamps, may minimize inter-day variability in fasting glucose but may also mask differences in bolus insulin requirements.

## 5. Conclusions

This article describes two studies that contribute to the growing body of evidence regarding optimizing mealtime insulin dosing in T1D. Our findings show there is little to no clinically, nor statistically significant postprandial glycemic impact of varying protein or carbohydrate type in the context of a mixed meal. Thus, these dietary factors likely do not need to be considered for evidence-based clinical guidelines or insulin dosing algorithms for T1D. Such guidelines of particular importance to reduce the risks associated with hyper- and hypoglycemia and to reduce the burden on individuals living with T1D.

## Figures and Tables

**Table 1:** Baseline demographics.

Demographic	Study 1: Protein Type (Mean ± SD)	Study 2: GI (Mean ± SD)
Total participants (*n*)	16	8
Women (*n*, %)	12 (75)	6 (75)
Age (years)	35.5 ± 18.2	29.0 ± 6.0
Body weight (kg)	74.4 ± 9.9	78.7 ± 18.5
BMI (kg/m^2^)	26.1 ± 3.8	26.2 ± 7.2
HbA1c (%,mmol/mol)	7.4 ± 1.2 (57 ± 11)	7.5 ± 0.7 (59 ± 8)
CRP (mg/L)	3.4 ± 5.8	3.2 ± 4.5
Total cholesterol (mmol/L)	5.0 ± 1.3	4.9 ± 1.3
Triglycerides (mmol/L)	0.9 ± 0.6	1.2 ± 1.2
HDL cholesterol (mmol/L)	1.7 ± 0.3	1.5 ± 0.3
LDL cholesterol (mmol/L)	2.8 ± 0.9	2.8 ± 0.7
C-Peptide (mmol/L)	Negative (<0.10)	Negative (<0.10)
T1D duration (years)	16.8 ± 9.6	18.0 ± 6.8
Total daily dose of insulin (insulin units/day)	48.0 ± 16.6	53.0 ± 11.7

GI: Glycemic Index; BMI: Body Mass Index; HbA1c: glycated hemoglobin; CRP: C-Reactive Protein; HDL: High Density Lipoprotein; LDL: Low Density Lipoprotein; T1D: type 1 diabetes.

**Table 2:** Glycemic outcome measures following egg, beef, chicken, salmon and whey protein mixed meals in adults with T1D, Study 1 (*n* = 16).

	Chicken	Egg	Beef	Salmon	Whey	Difference Between Groups (*p* Value)
5h iAUC (mmol·min/L)	203 ± 66	263 ± 100	309 ± 89	338 ± 83	397 ± 115	0.347
Early iAUC (0–2 h) (mmol/L)	92 ± 26	115 ± 43	148 ± 44	116 ± 38	181 ± 42	0.292
Late iAUC (2–5 h) (mmol·min/L)	133 ± 54	180 ± 76	201 ± 66	257 ± 66	281 ± 96	0.314
Fasting BGL (mmol/L)	7.4 ± 0.4	8.0 ± 0.6	7.6 ± 0.5	7.9 ± 0.5	8.6 ± 0.5	0.198
Incremental Mean BGL (mmol/L)	−0.5 ± 0.4	−0.3 ± 0.6	0.0 ± 0.5	0.3 ± 0.5	0.3 ± 0.5	0.380
SD BGL (mmol/L)	1.6 ± 0.2	1.7 ± 0.1	1.7 ± 0.2	1.5 ± 0.2	1.8 ± 0.1	0.480
CV (mmol/L)	25.3 ± 3.9	24.1 ± 2.7	23.4 ± 2.8	18.9 ± 2.4	21.7 ± 2.9	0.226
J-Index (mmol/L)	1.0 ± 0.4	2.4 ± 0.9	0.2 ± 0.1	2.2 ± 0.8	3.1 ± 1.1	0.028 *
Incremental Peak BGL (mmol/L)	2.2 ± 0.4	2.5 ± 0.5	2.7 ± 0.5	2.3 ± 0.5	3.0 ± 0.5	0.455
Time to Peak (minutes)	86 ± 23	74 ± 19	93 ± 23	133 ± 25	103 ± 21	0.313
Incremental Nadir BGL (mmol/L)	−2.5 ± 0.4	−2.6 ± 0.6	−2.2 ± 0.5	−2.1 ± 0.4	−2.3 ± 0.5	0.736
Time to Nadir (minutes)	118 ± 24	139 ± 25	148 ± 30	116 ± 28	142 ± 34	0.824
Amplitude (mmol/L)	4.7 ± 0.4	5.1 ± 0.4	4.9 ± 0.6	4.4 ± 0.5	5.3 ± 0.4	0.446
Incidence of Hypoglycemia (>3.5 mmol/L)	6/16	2/16	3/16	1/16	2/16	0.089
Mixed-modelling analysis	Factors affecting ‘5h iAUC’: Protein Type: *p* = 0.091 Fasting Mean BGL: *p* = 0.005 *					

iAUC: incremental area under curve; BGL: blood glucose level; SD: standard deviation; CV: coefficient of variation. Data are mean ± SEM values. * Statistically significant result (*p* < 0.05).

**Table 3:** Mean postprandial glycemic responses over 5 h to two high fat high protein meals, with either a low GI carbohydrate or a high GI carbohydrate in 8 adults with type 1 diabetes.

	High GI Carbohydrate Meal	Low GI Carbohydrate Meal	*p* Value
1 h iAUC (mmol/L∙min)	45 ± 19	28 ± 15	0.185
3 h iAUC (mmol/L∙min)	307 ± 128	260 ± 117	0.538
5 h iAUC (mmol/L∙min)	756 ± 188	692 ± 166	0.694
Mean absolute fasting BGL (mmol/L)	7.6 ± 0.4	7.9 ± 0.9	0.766
Incremental mean BGL (mmol/L)	2.1 ± 0.6	1.9 ± 0.5	0.646
Incremental SD (mmol/L)	2.0 ± 0.3	2.0 ± 0.3	0.881
Incremental peak BGL (mmol/L)	5.1 ± 0.8	4.9 ± 0.9	0.777
Time to peak BGL (min)	276 ± 18	263 ± 20	0.231
Nadir BGL (mmol/L)	−1.3 ± 0.2	−1.0 ± 0.2	0.250
Time to Nadir BGL (min)	4 ± 24	47 ± 22	0.100
Coefficient of Variation (%)	21 ± 4	20 ± 2	0.760
J-index	7.0 ± 2.5	6.4 ± 2.0	0.800
Incremental amplitude (mmol/L)	6.4 ± 0.8	5.9 ± 0.9	0.580
Incidence of hypoglycemia (<3.5 mmol/L)	0/8	0/8	N/A

Data are mean ± SEM unless otherwise indicated. GI: Glycemic Index, BGL: Blood Glucose Level; iAUC: incremental area under the curve.

## Data Availability

The original contributions presented in this study are included in the article/Supplementary Material. Further inquiries can be directed to the corresponding author.

## Supplementary Materials

The following supporting information can be downloaded at: https://www.mdpi.com/article/10.3390/nu17111868/s1, Table S1: Nutritional information for test meals in Study 1 (Protein); Table S2: Nutritional information for test meals in Study 2 (Glycemic Index); Figure S1: Risk of hypoglycemia following five protein sources (egg, beef, chicken, salmon and whey) with identical carbohydrate content in adults with type 1 diabetes (*n* = 16).
