# Supplementary material for: Do the Types of Dietary Carbohydrate and Protein Affect Postprandial Glycemia in Type 1 Diabetes?
Source: Nutrients. 2025 May 29;17(11):1868. doi: 10.3390/nu17111868 (PMC12158204; doi:10.3390/nu17111868)
Supplement: Supplementary file 1 [file nutrients-17-01868-s001.zip › nutrients-3654472-supplementary.pdf]

SUPPLEMENTARY MATERIAL

Li et al. Do The Types Of Dietary Carbohydrate And Protein Affect Postprandial Glycemia In Type 1 Diabetes?

Table S1: Nutritional information for test meals in Study 1 (Protein)

Test meal	Ingredients	Weight (g)	Energy (KJ (kcal))	Carbohydrate (g)	Protein (g)	Fat (g)
Egg	egg, scrambled	242 (raw)	2215 (530)	45	30	23
	white rice, cooked	104				
	mixed vegetables (peas, corn, carrot, green beans)	60				
	soy sauce	15				
Beef	lean beef blade steak, diced, grilled	137 (raw)	732 (175)	45	30	6
	white rice, cooked	104				
	mixed vegetables (peas, corn, carrot, green beans)	60				
	soy sauce	15				
Chicken	skin-free chicken breast, diced, grilled	135 (raw)	959 (229)	45	30	2
	white rice, cooked	104				
	mixed vegetables (peas, corn, carrot, green beans)	60				
	soy sauce	15				
Salmon	Atlantic salmon with skin, grilled	145 (raw)	627 (150)	45	30	19
	white rice, cooked	104				
	mixed vegetables (peas, corn, carrot, green beans)	60				
	soy sauce	15				
Whey	whey protein isolate, Isolyze, (protein powder)	36	1227 (294)	45	30	0
	white rice, cooked	104				
	mixed vegetables (peas, corn, carrot, green beans)	60				
	soy sauce	15				

Table S2: Nutritional information for test meals in Study 2 (Glycemic Index)

	Weight (g)	Energy (kJ (kcal))	CHO (g (%E))	Fat (g (%E))	Protein (g (%E))	Fiber (g)	GI (%)
**Low GI Carbohydrate Meal**							
Low GI, high fiber white bread	75	697	30	1	6	7	52 (Low)
Peanut butter	55	1430	7	29	13	-	N/A
**TOTAL**		**2127 (509)**	**37 (30)**	**29 (52)**	**19 (15)**	**7**	
**High GI Carbohydrate Meal**							
High GI, high fiber white bread	74	738	30	3	6	5	76 (High)
Peanut butter	55	1430	7	28	13	-	N/A
**TOTAL**		**2168 (519)**	**37 (29)**	**31 (55)**	**19 (15)**	**5**	

CHO, Carbohydrate; %E, Percentage of total energy in the meal; GI, Glycemic Index.

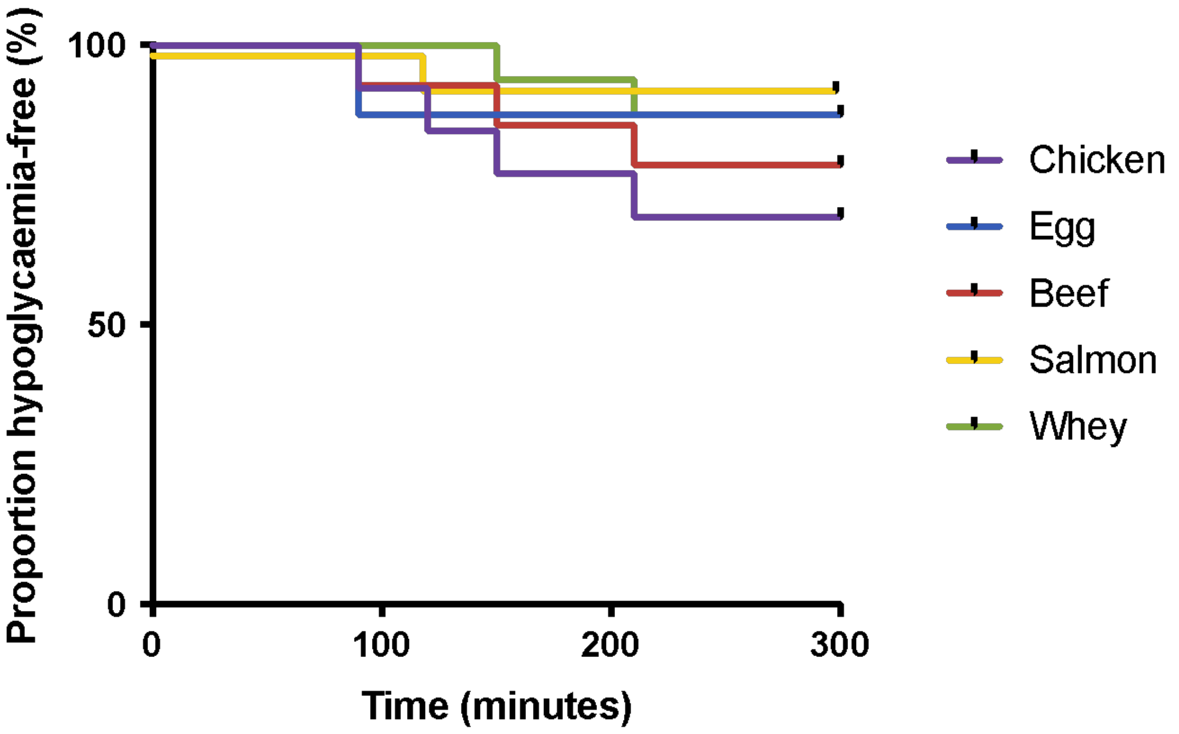

Figure S1: Risk of hypoglycemia following five protein sources (egg, beef, chicken, salmon and whey) with identical carbohydrate content in adults with type 1 diabetes (n = 16)
